# Self-report of medical diagnosis of chronic kidney disease: prevalence and characteristics in the Brazilian adult population, National Health Survey 2013 and 2019

**DOI:** 10.1590/SS2237-9622202200017.especial

**Published:** 2022-08-01

**Authors:** Ellen de Cassia Dutra Pozzetti Gouvêa, Celia Landmann Szwarcwald, Giseli Nogueira Damacena, Lenildo de Moura

**Affiliations:** 1 Ministério da Saúde, Departamento de Análise Epidemiológica e Vigilância de Doenças não Transmissíveis, Brasília, DF, Brazil; 2 Fundação Instituto Oswaldo Cruz, Instituto de Comunicação e Informação Científica e Tecnológica em Saúde, Rio de Janeiro, RJ, Brazil; 3 Fundação Instituto Oswaldo Cruz, Rio de Janeiro, RJ, Brazil; 4 Organização Pan-Americana da Saúde na Bolívia, Unidade Técnica de Doenças Crônicas Não Transmissíveis e Saúde Mental, Cidade de La Paz, Estado de La Paz, Bolivia

**Keywords:** Renal Insufficiency Chronic, Risk Factors, Prevalence, Health Surveys

## Abstract

**Objective:**

To estimate the prevalence of chronic kidney disease (CKD) in the adult Brazilian population and to describe its characteristics, according to the National Health Survey (PNS) 2013-2019.

**Methods:**

Descriptive cross-sectional study, with adults participating in the PNS, based on self-reported medical diagnosis of CKD. Prevalence of CKD and their respective 95% confidence intervals (95%CI) were estimated for Brazil.

**Results:**

In 2013, 60,202 individuals were analyzed, and in 2019, 85,854. The prevalence of self-reported diagnosis of CKD in both editions was 1.4% and increased with increasing age. In 2019, the prevalence of self-reported CKD was 3.3% (95%CI 2.9;3.7) in hypertensive individuals, 4.1% (95%CI 3.4;5.0) among diabetics, and 3.3% (95%CI 2.8;3.9) in those reporting hypercholesterolemia.

**Conclusion:**

The prevalence of CKD in Brazil remained stable in the period but reinforces the need for expansion of diagnosis and strengthening of primary care in the Brazilian National Health System (SUS).

**Study contributions**

**Main results**

The prevalence of self-reported diagnosis of chronic kidney disease (CKD), for the 2013 and 2019 editions of the National Health Survey, was 1.4%, with 1.3% in males and 1.5% in females.

**Implications for services**

CKD is an outcome that can be avoided with strategies that address comorbidities and risk factors. Strengthening primary care and interventions that slow the progression of these events can be effective measures.

**Perspectives**

Considering the impact of CKD on the quality of life of individuals, population ageing and the high cost of treatment, it is necessary to plan how to tackle the burden of this disease in Brazil.

## Introduction

Chronic kidney disease (CKD), defined as a heterogeneous group of diseases that affect the structure and function of the kidneys for more than three months,^1^ presents significant morbidity and mortality (often premature), reduces the quality of life,^2^ and its burden has increased worldwide. The increase in prevalence is associated with population ageing and diseases such as hypertension and diabetes *mellitus*.^3^ ^,^ ^4^

Approximately 10% of the world population has CKD,^5^ however, prevalence estimates vary both within and between countries, either due to the approach used to assess, define and diagnose the disease, or owing to individual and sociodemographic factors. CKD also generates socioeconomic impacts on account of the high cost of treatment and the limitations it imposes on daily life activities.^6^

CKD is associated with the development of cardiovascular diseases (CVD), as well as they share several risk factors,^5^ ^,^ ^7^ including obesity, smoking, hypertension, hypercholesterolemia and diabetes *mellitus*.^3^ The main cause of mortality among CKD people with is CVD,^8^ moreover, the risk of CVD in an individual with CKD is higher than in those without the disease.^9^

A report by the Brazilian Society of Nephrology showed a 58% increase in the prevalence of individuals on chronic dialysis in Brazil between 2009 and 2018, a 20% increase in the incidence rate and a slight increase (from 17.1% to 19.5%) in the gross mortality rate between 2013 and 2018.^10^ The monitoring of such data contributes to the implementation of assistance policies for people with CKD^10^ through strategies such as multidisciplinary approaches and educational actions for the population.^10^

The National Health Survey (PNS) makes it possible to know the magnitude of several diseases, including CKD, based on precise indicators.^11^ Considering that this disease has contributed to the morbidity and mortality of the Brazilian population,^10^ this study aimed to estimate its prevalence in the Brazilian adult population and to describe its characteristics based on data collected on the 2013 and 2019 editions of the PNS.

## Methods

This is a descriptive cross-sectional study that used data from two editions of the PNS, 2013 and 2019, as sources of information, whose databases were accessed on the website of the Brazilian Institute of Geography and Statistics (IBGE) (https://bit.ly/3KxbMMu), in March 2021.

The PNS is a nationwide population-based household survey, carried out by the Ministry of Health in partnership with the IBGE, as part of the Integrated Household Surveys System. The sample was selected by clusters in three stages, with stratification of the primary sampling units, whose procedures have been previously reported.^12^ In both editions of the PNS, fieldwork was carried out by the IBGE and, for the interviews, mobile collection devices were used. The questionnaire was subdivided into three parts: household; all residents of the household; and selected individual. The first and the second parts of the questionnaire were answered by a resident who knew how to inform about the socioeconomic and health situation of all the other residents. The third part was answered by the selected resident with equiprobability among all residents of the household.^11^ ^,^ ^12^ More information about the PNS can be found in previous publication.^11^

In 2013, individuals aged ≥ 18 years were selected, and in 2019, individuals aged ≥ 15 years, residents of permanent private households in Brazil. The present study only includes individuals aged ≥ 18 years in order to allow comparison between the two editions.

To calculate the prevalence of self-reported CKD in the Brazilian population, the following question was used: *Has any doctor ever diagnosed you with chronic kidney failure?*, with “yes/no” as response options.

The prevalence of self-reported CKD was analyzed according to sociodemographic characteristics: sex (male; female); age group (in complete years, 18-29; 30-44; 45-59; 60-74; ≥ 75); self-reported race/skin color (White; Brown; Black); education (no schooling/incomplete elementary school; incomplete high school; complete high school and over) and per capita income (less than one minimum wage - MW; between one and two MWs; two or more MWs). 

For the characterization of CKD, the following variables were considered, among those with a self-reported diagnosis of CKD: mean age at the first diagnosis of CKD; kidney transplantation due to CKD (yes; no); hemodialysis due to CKD (yes; no); peritoneal dialysis due to CKD (yes; no); takes medication due to CKD (yes; no); regular follow-up with a health professional due to CKD (yes; no) - only for 2019, as this question was not included in the 2013 PNS questionnaire; degree of limitation of usual activities due to CKD (does not limit; a little; moderately; intensely/very intensely).^13^ ^,^ ^14^

Furthermore, the prevalence of self-reported CKD according to a self-reported diagnosis of hypertension, diabetes and high cholesterol was investigated, using the questions: *Has any doctor ever diagnosed you with hypertension? Has any doctor ever diagnosed you with diabetes?* and *Has any doctor ever diagnosed you with high cholesterol?*, with binary response options (yes; no). Diagnoses of hypertension and diabetes during pregnancy were excluded. Additionally, data related to age groups (in years) of the first diagnosis for hypertension and diabetes (18 to 39; 40 to 59; ≥ 60), and regular medical/health service appointments due to hypertension or diabetes (yes, regularly; no, only in the event of a problem; never visits a doctor for follow-ups) was also considered.

The prevalence of self-reported CKD was also investigated according to the consumption of tobacco: smoker, defined based on the question *Do you currently smoke any tobacco product?* (yes, daily, and yes, less than daily); former smoker, based on the question: *And in the past, did you use to smoke any tobacco product?* (yes, daily and yes, less than daily); never smoked, according to the answers: I do not currently smoke, and No, I never smoked for the previous questions and, among former smokers, how long they smoked: up to 5 years; from 6 to 10 years; from 11 to 20 years; for ≥ 21 years.

In relation to obesity, in the 2013 PNS, the body mass index (BMI) was used, calculated from weight and height measured by dividing the value of body mass, in kilograms, by the square of height, in meters, and obesity was defined by BMI values greater than or equal to 30 kg/m^2^. In 2019, anthropometric measurements were performed only on a subsample of insufficient size for the present study.

In the data analysis, the prevalence and the respective 95% confidence intervals (95%CI) were estimated. Data from the two editions of the PNS were analyzed using the statistics package IBM SPSS Statistics - version 21 (IBM, 2012) through the complex sample module, considering the sampling design, including expansion factors and clustering effects.^15^

The expansion factors, which correspond to the inverse of the product of the selection probabilities at each stage, including a correction factor for losses, were calibrated taking into account population projections for Brazil and the Units of the Federation.^12^ To enable comparisons between both editions of the PNS, the IBGE carried out a new calibration of the expansion factors of the 2013 PNS, considering the revision of the Population Projection of the Units of the Federation by sex and age for the period 2010-2060, released in 2018. This same population projection was used in the calibration of the weights of the 2019 PNS, thus ensuring comparability between the two editions of the survey.

The 2013 PNS was approved by the National Committee for Ethics in Research (Comissão Nacional de Ética em Pesquisa - CONEP) in July, 2013 under No. 328,159, and the 2019 PNS in August, 2019, under No. 3,529,376.

## Results

In 2013, information from 60,202 individuals aged 18 years or older was analyzed and, in 2019, from 85,854 individuals in the same age group. The prevalence of self-reported diagnosis of CKD, for both editions of the PNS, was 1.4% (95%CI 1.3;1.6), with 852 respondents reporting CKD in 2013, and 1,261 in 2019. Among males, the prevalence was 1.3% (95%CI 1.1;1.6, in 2013, and 95%CI 1.2;1.5, in 2019). Among females, the prevalence was 1.5% (95%CI 1.3;1.7) in both editions. Higher prevalence was observed among those aged ≥ 75 years, both in 2013 and 2019 (3.4%; 95%CI 2.2;5.2 and 3.1%; 95%CI 2.5;3.9, respectively), and among people who declared themselves White (Table 1).

**Table 1:** Prevalence of self-reported chronic kidney disease (CKD) and sociodemographic characteristics among individuals aged ≥ 18 years, National Health Survey, Brazil, 2013 and 2019

Sociodemographic characteristics	2013 (n = 60,202)			2019 (n = 85,854)		
	n	%	95%CI^a^	n	%	95%CI^a^
Total among individuals with a diagnosis of CKD	852	1.4	1.3;1.6	1.261	1.4	1.3;1.6
**Sex**						
Male	381	1.3	1.1;1.6	553	1.3	1.2;1.5
Female	470	1.5	1.3;1.7	708	1.5	1.3;1.7
**Age group**						
18 to 29	82	0.5	0.4;0.7	133	0.7	0.5;1.0
30 to 44	167	0.9	0.7;1.1	253	1.0	0.8;1.2
45 to 59	301	2.0	1.7;2.4	402	1.8	1.5;2.1
60 to 74	209	2.6	2.0;3.2	325	2.4	2.0;2.8
≥ 75	93	3.4	2.2;5.2	148	3.1	2.5;3.9
**Race/skin color**						
White	449	1.6	1.3;1.8	611	1.6	1.4;1.8
Brown	304	1.2	1.0;1.4	129	1.3	1.1;1.5
Black	85	1.5	1.0;2.3	504	1.2	0.9;1.7
**Education**						
No schooling/incomplete elementary education	479	2.0	1.7;2.4	592	2.0	1.7;2.3
Incomplete high school	110	1.2	0.9;1.5	214	1.7	1.3;2.2
Complete high school and over	262	1.0	0.8;1.2	455	1.0	0.9;1.2
***Per capita* income (minimum wages)**						
< 1	597	1.4	1.3;1.6	656	1.4	1.2;1.6
1-2	175	1.5	1.2;1.9	344	1.5	1.3;1.8
> 2	80	1.1	0.8;1.6	261	1.4	1.1;1.7

a) 95%CI: 95% confidence intervals

Individuals with no education or with incomplete elementary education had the highest prevalence of self-reported CKD, 2.0% (95%CI 1.7;2.4 in 2013, and 95%CI 1.7;2.3, in 2019). Additionally, those mostly affected by CKD had a *per capita* income between one and two MWs, for both years (Table 1).

The variables referring to the characterization of self-reported CKD, in 2013 and 2019, are presented in Table 2. The mean age at the first self-reported diagnosis of CKD was 40.2 (95%CI 38.1;42.2) in 2013, and 38.8 (95%CI 36.8;40.7) in 2019. Among renal replacement therapies, the prevalence of kidney transplantation was 2.0% (95%CI 1.1;3.6) in 2013, and 3.9% (95%CI 2.1;7.1) in 2019; the prevalence of hemodialysis was 7.4% (95%CI 4.9;11.0) in 2013 and 5.6% (95%CI 4.1;7.6) in 2019, and prevalence of peritoneal dialysis was 1.5% (95%CI 0.8;3.0) in 2013, and 2.5% (95%CI 1.3;5.0) in 2019. In terms of taking medication for CKD, there was a reduction from 57.9% (95%CI 52.3;63.2) to 49.4% (95%CI 44.7;54.0), between 2013 and 2019. Regular monitoring of CKD with a health professional could only be analyzed in 2019: 56.3% (95%CI 51.6;60.8) of the individuals diagnosed with the disease were under regular monitoring. With regard to limitation of usual activities, more than 30% of the participants with CKD reported some degree of limitation in both editions.

**Table 2:** Self-reported prevalence of chronic kidney disease (CKD), characteristics, complications, and care among individuals aged ≥ 18 years, National Health Survey, Brazil, 2013 and 2019

Characterization of the CKD, complications and care		2013 (n = 856)			2019 (n = 1,332)		
		n	%	95%CI^a^	n	%	95%CI^a^
**Chronic kidneyt disease**		852	1.4	1.3;1.6	1,261	1.4	1.3;1.6
**Average age at first diagnosis of CKD**		-	40.2 SD^b^ 1.055	38.1;42.2	-	38.8 SD^b^ 0.994	36.8;40.7
**Kidney transplant due to CKD**	Yes	17	2.0	1.1;3.6	50	3.9	2.1;7.1
	No	835	98.0	96.4;98.9	1,211	96.1	92.9;97.9
**Hemodialysis due to CKD**	Yes	63	7.4	4.9;11.0	72	5.6	4.1;7.6
	No	789	92.6	89.0;95.1	1,189	94.4	92.4;95.9
**Peritoneal dialysis due to CKD**	Yes	13	1.5	0.8;3.0	33	2.5	1.3;5.0
	No	839	98.5	97.0;99.2	1,228	97.5	95.0;98.7
**Takes medications due to CKD**	Yes	493	57.9	52.3;63.2	658	49.4	44.7;54.0
	No	359	42.1	36.8;47.7	674	50.6	46.0;55.3
**Has regular follow-ups with a health professional due to CKD**	Yes	-	-	-	705	56.3	51.6;60.8
	No	-	-	-	556	43.7	39.2;48.4
**Degree of limitation of daily life activities due to CKD**	No limit	507	59.5	54.1;64.7	759	60.0	55.4;64.4
	A little	168	19.8	15.9;24.3	215	17.6	14.6;21.2
	Moderately	No limit	9.1	6.8;12.1	134	10.4	7.7;13.8
	Intense/Really intense	99	11.7	8.5;15.7	153	12.0	9.4;15.1

a) 95%CI: 95% confidence intervals; b) SD: Standard deviation.

The prevalence of self-reported CKD among individuals diagnosed with hypertension was higher than among individuals without a diagnosis of this comorbidity [2.9% (95%CI 2.4;3.4) in 2013, and 3.3% (95%CI 2.9;3.7) in 2019]. In the 2013 edition, in relation to the age at the first diagnosis of hypertension, for 3.2% (95%CI 1.9;5.3) of the individuals it was at the age of 60 or over. In 2019, 3.7% (95%CI 2.3;4.1) of the diagnoses occurred between 18 and 39 years of age (Table 3).

**Table 3:** Prevalence of self-reported diagnosis of hypertension among individuals aged ≥ 18 years with self-reported diagnosis of chronic kidney disease (CKD), National Health Survey, Brazil, 2013 and 2019

Hypertension		2013 (n = 856)			2019 (n = 1,332)		
		n	%	95%CI^a^	n	%	95%CI^a^
**Hypertension**	Yes	368	2.9	2.4;3.4	694	3.3	2.9;3.7
	No	484	1.0	0.9;1.2	638	0.9	0.8;1.0
**Age at first diagnosis of hypertension**	18 to 39	107	2.8	2.1;3.7	221	3.7	2.9;4.7
	40 to 59	168	2.7	2.1;3.4	304	2.8	2.4;3.4
	≥ 60	68	3.2	1.9;5.3	115	3.1	2.3;4.1
**Visits a doctor/health care service regularly due to hypertension**	Yes, regularly	259	3.4	2.7;4.2	482	3.8	3.2;4.5
	No, only in the event of a problem	79	2.0	1.4;2.7	177	2.7	2.1;3.5
	Never visits a doctor for follow-ups	30	2.5	1.4;4.5	34	1.6	1.0;2.6

a) 95%CI: 95% confidence intervals.

In 2013, among those with diabetes, the prevalence of self-reported CKD was 3.4% (95%CI 2.6;4.5) and, in 2019, it was 4.1% (95%CI 3.4;5.0). Regarding the age group of the first diagnosis of diabetes, in 2013, individuals who received the diagnosis of diabetes at the age of 60 or older had a prevalence of self-reported CKD of 3.7% (95%CI 2.0;6.5), and in 2019, of 4.5% (95%CI 3.1;6.4). Regarding regular medical appointments (or visits to the healthcare service) due to diabetes, the prevalence of self-reported CKD was 3.5% (95%CI 2.5;5.0) in 2013, and 4.5% (95%CI 3.6;5.6), in 2019 (Table 4).

**Table 4:** Prevalence of self-reported diagnosis of diabetes among individuals aged ≥ 18 years diagnosed with chronic kidney disease (CKD), National Health Survey, Brazil, 2013 and 2019

Diabetes		2013 (n = 856)			2019 (n = 1,332)		
		n	%	95%CI^a^	n	%	95%CI^a^
**Diabetes**	Yes	129	3.4	2.6;4.5	282	4.1	3.4;5.0
	No	773	1.3	1.1;1.5	1,050	1.2	1.1;1.3
**Age at first diagnosis of diabetes**	18 to 39	13	2.3	1.4;4.0	42	3.6	2.4;5.4
	40 to 59	66	3.3	2.3;4.8	147	4.1	3.1;5.3
	≥ 60	37	3.7	2.0;6.5	83	4.5	3.1;6.4
**Visits a doctor/health care service regularly due to diabetes**	Yes, regularly	90	3.5	2.5;5.0	222	4.5	3.6;5.6
	No, only in the event of a problem	27	3.1	1.8;5.5	42	3.0	1.9;4.6
	Never visits a doctor for follow-ups	13	3.8	1.7;7.7	19	3.3	1.7;6.4

a) 95%CI: 95% confidence intervals.

For this study, obesity was analyzed only in the 2013 edition of the PNS, with a prevalence of self-reported CKD of 1.5% (95%CI 1.2;1.9) among individuals identified as obese. For individuals with hypercholesterolemia, the prevalence of self-reported CKD was 3.6% (95%CI 3.0;4.3) in 2013, and 3.3% (95%CI 2.8;3.9) in 2019 (Table 5).

**Table 5:** Prevalence of obesity, self-reported diagnosis of high cholesterol, and tobacco smoking among individuals aged ≥ 18 years with a self-reported diagnosis of chronic kidney disease (CKD), National Health Survey, Brazil, 2013 and 2019

Diabetes		2013 (n = 856)			2019 (n = 1,332)		
		n	%	95%CI^a^	n	%	95%CI^a^
**Obesity^b^**	Yes	190	1.5	1.2;1.9	-	-	-
	No	662	1.4	1.2;1.6	-	-	-
**Diagnosis of high cholesterol**	Yes	268	3.6	3.0;4.3	425	3.3	2.8;3.9
	No	527	1.2	1.0;1.4	797	1.2	1.0;1.3
**Tobacco smoking**	Smoker	172	2.0	1.5;2.5	156	1.4	1.1;1.9
	Former smoker	240	2.3	1.8;2.8	432	1.8	1.6;2.1
	Never smoked	440	1.1	0.9;1.3	673	1.2	1.1;1.4
**Among former smokers, how long the individual was a smoker (years)**	For up to 5	11	1.0	0.4;2.4	30	1.0	0.6;1.6
	From 6 to 10	15	1.4	0.7;2.8	33	1.3	0.9;2.0
	From 11 to 20	52	2.5	1.7;3.6	76	1.7	1.2;2.5
	≥ 21 years	128	3.3	2.4;4.7	228	2.8	2.3;3.4

a) 95%CI: 95% confidence intervals; b) For the year 2019, it was not possible to estimate the prevalence of chronic kidney disease (CKD) according to obesity, because, in that edition, anthropometric measurements were performed only on a subsample, whose size was insufficient to estimate a disease which does not have high prevalence in the population, such as CKD.

The prevalence of self-reported CKD in former smokers was 2.3% (95%CI 1.8;2.8) in 2013, and 1.8% (95%CI 1.6;2.1) in 2019 and, among them, a gradient in the prevalence of CKD can be observed: the longer the individual's exposure to tobacco, the higher the prevalence, reaching 3.3% (95%CI 2.4;4.7) and 2.8% (95%CI 2.3;3.4) among those who smoked for ≥ 21 years or more, in 2013 and 2019, respectively (Table 5).

## Discussion

The prevalence of CKD remained stable from 2013 to 2019. Higher prevalence was found in the elderly, individuals who declared themselves to be of White race/skin color, and with a lower level of education and income.

It is worth noting that the PNS uses self-reported data from the population, therefore, the 1.4% total prevalence for the Brazilian adult population, in both editions, may be underestimated. In an analysis performed on a subsample of the 2013 PNS, based on laboratory data, the estimate of CKD was up to four times higher when compared to the self-reported prevalence.^19^

The prevalence of CKD found in the PNS was about 10 percentage points lower when compared to national^16^ ^,^ ^17^ and international literature.^18^ ^-^ ^21^ In a survey with biological samples, carried out between 2007 and 2009 in Canada, the prevalence of CKD was 12.5%, representing about 3 million Canadian adults.^18^ Also with laboratory data, in the United States between 2015 and 2018, and China between 2009 and 2010, the prevalence among adults was greater than 10%.^19^ ^,^ ^20^ Corroborating this finding, a systematic review and meta-analysis of observational studies, which aimed to determine the overall prevalence of CKD in the general adult population, according to the Kidney Outcomes Quality Initiative criteria, found an overall prevalence of CKD between 11% and 13%.^2^

Considering that CKD is an outcome that can be avoided with strategies that address risk factors such as diabetes *mellitus* and hypertension, establishing interventions that delay the progression of these events into more severe form, taking into account the ageing of the population, can be an effective measure.

In the 2019 PNS, the prevalence of self-reported CKD was almost five times higher in individuals aged ≥ 75 years, when compared to those aged 18 to 29 years. Corroborating this finding, data from the Centers for Disease Control and Prevention in the United States show that CKD was present in 38% of individuals aged ≥ 65 years.^19^ The increase in prevalence associated with ageing was found in several studies, given that physiological changes such as a decrease in estimated glomerular filtration rate (a marker of kidney damage), naturally occur with advancing age.^14^ ^,^ ^22^ ^,^ ^23^ This condition may suggest that individuals are receiving better quality care, and that there has been improvement in dialysis techniques and medications to support CKD complications and, therefore, survival has increased, which, as a consequence, reflects into a higher prevalence in those of a more advanced age.

Regarding socioeconomic aspects, higher prevalence of CKD in individuals with lower levels of education and income was observed in both editions of the PNS. Similarly, other studies emphasize the socioeconomic inequalities of CKD.^5^ In a systematic review and meta-analysis conducted with 35 studies published up to January 2013 in Medline and Embase, social and economic disparities were strongly associated with CKD,^24^ both for incident chronic kidney disease, and the faster progression of such disease.^25^ In addition, other risk factors may also be present, such as food insecurity and greater difficulty in accessing health care. Therefore, such data reinforce that accessibility is an important barrier to be faced in order to reduce inequalities in the treatment of CKD.

Other factors analyzed in the present study, related to access to health care, were: age at diagnosis of CKD and the proportions of individuals who underwent kidney transplantation, whether they were on hemodialysis or peritoneal dialysis, whether they were using medication for CKD and whether there was regular follow-up with a health professional (the latter valid only for 2019). The analysis showed the need for good governance in the health system, with the organization of the comprehensive care line, provided for in Ordinance No. 1,168/GM of June 15, 2004, which established the National Policy for the Care of Patients with Kidney Disease.^13^ Despite the efforts undertaken to reduce chronic conditions in Brazil, challenges still need to be overcome to ensure improved care for people with CKD.^13^

In the present study, more than a third of individuals with CKD reported some degree of limitation in their usual activities, in both editions of the PNS. Corroborating this, a cross-sectional study with individuals on hemodialysis showed that the quality of life was impaired in 31.5% of the investigated population.^26^ This limitation may be related to the time and routine dedicated to the treatment, decreased physical performance, symptoms of the disease and late diagnosis.

Data from the 2018 Brazilian Dialysis Census showed hypertension as the main underlying cause, followed by diabetes.^10^ ^,^ ^14^ According to PNS data, in Brazil, individuals with hypertension had a higher prevalence of CKD when compared to those without a diagnosis of hypertension, a result that is consistent with data from Canada,^18^ but different from what was observed in Africa and Thailand.^21^ ^,^ ^23^ In the United States, approximately one in three adults with diabetes *mellitus* and one in five with hypertension had CKD.^19^

Obesity has been recognized as an important cause and cofactor in the development and progression of CKD,^27^ as have high cholesterol and tobacco smoking. However, in the PNS, the prevalence of CKD among individuals with obesity, who reported having hypercholesterolemia and who smoked tobacco products was lower than that what was found in Thailand.^22^ Such data draw attention to the need for more specific studies of risk factors associated with CKD in Brazil.

Former smokers who had greater exposure to tobacco (> 21 years) had a higher prevalence of self-reported CKD, but it is not possible to infer whether smoking cessation occurred before or after the diagnosis of CKD. However, it is worth highlighting the importance of the policies for tobacco smoking reduction, so successful in Brazil.

The results of this study should be considered within the context of its limitations. As this is a cross-sectional study, we cannot describe causal relationships, nor establish the chronology of events. Additionally, as these are self-reported conditions, there may have been an underestimation of prevalence, since studies using laboratory measurements showed a higher prevalence.^19^

The estimated prevalence of CKD in Brazil remained stable between 2013 and 2019. However, the prevalent cases tend to increase with the ageing of the Brazilian population, which reinforces the need for greater attention to health promotion and healthy ageing, particularly with regard to social determinants. The strengthening of primary healthcare in the Brazilian National Health System (SUS), as a public health priority, and strict compliance with the guidelines for the treatment of hypertension and diabetes *mellitus* would already be relevant measures for fighting CKD. Furthermore, with the high cost of renal replacement therapies (hemodialysis, peritoneal dialysis and kidney transplantation), for which the SUS is the largest funder in Brazil, there is a need for prioritization and strategic planning to deal with the burden of this disease.

Considering the potential underestimation of prevalence, the high cost of treatment, population ageing and the impact of CKD on quality of life, it is suggested that more specific epidemiological studies with laboratory data be conducted.
